# COM1/348: Design and Implementation of a Portal for the Market of the Medical Equipment (MEDICOM)

**DOI:** 10.2196/jmir.1.suppl1.e14

**Published:** 1999-09-19

**Authors:** S Palamas, I Vlachos, O Panou-Diamandi, G Marinos, D Kalivas, C Zeelenberg, C Nimwegen, D Koutsouris

**Affiliations:** ^1^National Technical University of AthensAthensGreece

**Keywords:** Medical Devices, Electronic Commerce

## Abstract

**Introduction:**

The MEDICOM system provides the electronic means for medical equipment manufacturers to communicate online with their customers supporting the Purchasing Process and the Post Market Surveillance. The MEDICOM service will be provided over the Internet by the MEDICOM Portal, and by a set of distributed subsystems dedicated to handle structured information related to medical devices. There are three kinds of these subsystems, the Hypermedia Medical Catalogue (HMC), Virtual Medical Exhibition (VME), which contains information in a form of Virtual Models, and the Post Market Surveillance system (PMS). The Universal Medical Devices Nomenclature System (UMDNS) is used to register all products. This work was partially funded by the ESPRIT Project 25289 (MEDICOM).

**Methods:**

The Portal provides the end user interface operating as the MEDICOM Portal, acts as the yellow pages for finding both products and providers, providing links to the providers servers, implements the system management and supports the subsystem database compatibility. The Portal hosts a database system composed of two parts: (a) the *Common Database*, which describes a set of encoded parameters (like Supported Languages, Geographic Regions, UMDNS Codes, etc) common to all subsystems and (b) the *Short Description Database*, which contains summarised descriptions of medical devices, including a text description, the codes of the manufacturer, UMDNS code, attribute values and links to the corresponding HTML pages of the HMC, VME and PMS servers. The Portal provides the MEDICOM user interface including services like end user profiling and registration, end user query forms, creation and hosting of newsgroups, links to online libraries, end user subscription to manufacturers' mailing lists, online information for the MEDICOM system and special messages or advertisements from manufacturers.

**Results:**

Platform independence and interoperability characterise the system design. A general purpose RDBMS is used for the implementation of the databases. The end user interface is implemented using HTML and Java applets, while the subsystem administration applications are developed using Java. The JDBC interface is used in order to provide database access to these applications. The communication between subsystems is implemented using CORBA objects and Java servlets are used in subsystem servers for the activation of remote operations.

**Discussion:**

In the second half of 1999, the MEDICOM Project will enter the phase of evaluation and pilot operation. The benefits of the MEDICOM system are expected to be the establishment of a world wide accessible marketplace between providers and health care professionals. The latter will achieve the provision of up-to-date and high quality products information in an easy and friendly way, and the enhancement of the marketing procedures and after sales support efficiency.

